# Staphylococcal Enterotoxin B and C Mutants and Vaccine Toxoids

**DOI:** 10.1128/spectrum.04446-22

**Published:** 2023-02-23

**Authors:** Patrick M. Schlievert

**Affiliations:** a Department of Microbiology and Immunology, Carver College of Medicine, University of Iowa, Iowa City, Iowa, USA; Yale University

**Keywords:** *Staphylococcus aureus*, vaccination, staphylococcal enterotoxins, superantigens, enterotoxin, pyrogenic toxin, toxoid, vaccines

## Abstract

Three mutants individually of both staphylococcal enterotoxins B and C were prepared by site-specific mutagenesis of enterotoxin amino acids that contact host T lymphocyte immune cell receptor sites (N23A, Q210A, and N23A/Q210A); these amino acids are shared between the two enterotoxins, and mutations reduce the interaction with the variable part of the β-chain of the T lymphocyte receptor. The mutant proteins, as expressed in Staphylococcus aureus RN4220, lacked biological toxicity as measured by the loss of (i) stimulation of rabbit splenocyte proliferation, (ii) pyrogenicity, and (iii) the ability to enhance the lethality of endotoxin shock, compared to wild-type enterotoxins. In addition, the mutants were able to vaccinate rabbits against pyrogenicity, the enhancement of endotoxin shock, and lethality in a pneumonia model when animals were challenged with methicillin-resistant S. aureus. Three vaccine injections (one primary and two boosters) protected rabbits for at least 3.5 months postvaccination when challenged with wild-type enterotoxins (last time point tested). These mutant proteins have the potential to function as toxoid vaccines against these two causes of nonmenstrual toxic shock syndrome (TSS).

**IMPORTANCE** Toxic shock syndrome toxin 1 (TSST-1) and staphylococcal enterotoxins B and C cause the majority of cases of staphylococcal toxic shock syndrome. Previously, vaccine toxoids of TSST-1 have been prepared. In this study, vaccine toxoids of enterotoxins B and C were prepared. The toxoids lost biological toxicity but were able to vaccinate rabbits against lethal TSS.

## INTRODUCTION

There are three major pyrogenic toxin superantigens associated with the causation of staphylococcal toxic shock syndrome (TSS). These include TSS toxin 1 (TSST-1) and staphylococcal enterotoxins (SEs) B (SEB) and C (SEC) (1, 2). TSST-1 causes 100% of menstrual, vaginally associated TSS cases and 50% of nonmenstrual TSS cases (3). When TSST-1 is present in postinfluenza nonmenstrual TSS, the infection is nearly universally fatal (4). SEB and SEC are associated with nearly 50% of nonmenstrual TSS cases (5). Occasionally, other pyrogenic toxin superantigens, for example, enterotoxin A, are associated with nonmenstrual TSS cases (6).

Staphylococcal pyrogenic toxin superantigens are well known to cross-bridge the variable part of the β-chain of the T lymphocyte receptor (Vβ-TCR) with the α- and/or β-chain of major histocompatibility complex class II (MHC II) molecules (1, 2). This cross-bridging leads to the activation and proliferation of a large percentage (up to 50%) of T cells and the activation of antigen-presenting cells, with the latter most often viewed as macrophage activation. The net effect is massive cytokine production, or the first-described so-called cytokine storm, with interleukin-1β causing high fever (≥102°F) and tumor necrosis factor alpha (TNF-α) and TNF-β causing capillary leak and consequent hypotension (systolic blood pressure of <90 mm Hg), shock, and possibly death (1, 2).

There are other superantigen sites identified for interactions with human cell receptors. These are the emetic cystine loop on SEs, not present on other superantigens (1, 2), and CD40 and gp130 interaction sites that are not well characterized but lead to the activation of epithelial cells (CD40) (7), keratinocytes (CD40 and gp130) (8), and adipocytes (gp130) (9) to produce cytokines.

The amino acids on TSST-1, SEB, and SEC that are required for the activation of T cells and macrophages have been localized through three-dimensional complex structure determinations combined with mutagenesis of individual amino acids with loss of superantigenicity (1, 2). For example, in the standard view (O/B fold to the right and β-grasp to the left), amino acids, including G31S/S32P (glycine at amino acid position 31 changed to serine and serine at position 32 changed to proline) in the O/B fold of TSST-1, are required for interactions with MHC II molecules (2, 10). The same general region on SEB and SEC (for example, amino acid residue D45) is also involved in MHC II interactions, with these two superantigens sharing the required amino acids but not sharing residues with those required for TSST-1 interaction with MHC II (1, 2, 11).

SEB and SEC also share amino acids required for the Vβ-TCR interaction in a groove at the top of the front standard view between the O/B fold and β-grasp domains (1, 2, 11). In contrast, the domain for TSST-1 interactions with Vβ2-TCR, the only interacting TCR for TSST-1, is along the exposed diagonal α-helix on the back side of the standard pyrogenic toxin superantigen view (including amino acids H135, Q136, and Q139) (1, 2, 10). This means that in the complex structures, SEB and SEC form wedges along the side of the MHC II-TCR complex, whereas TSST-1 forms a structure consistent with three beads on a string (1, 2).

There are three predominant measurable activities of pyrogenic toxin superantigens, as studied *in vivo* (pyrogenicity and enhancement of endotoxin [lipopolysaccharide {LPS}] shock) (12, 13) and *in vitro* (superantigenicity) (14). Prior to being referred to as superantigens, this large family of exotoxins was referred to as pyrogenic toxins (15). In this study, the family is referred to as pyrogenic toxin superantigens to reflect the above-mentioned activities.

The biological activities of pyrogenic toxin superantigens are most easily seen with the use of rabbits and their splenocytes. The fever response of the toxins typically requires 1.0 to 5.0 μg/kg of body weight to cause significant fevers in rabbits, peaking 4 h after intravenous (i.v.) administration; fevers as high as 2.0°C can be seen when 100 μg/kg pyrogenic toxin superantigen is administered by this route (12). The enhancement of endotoxin shock can be as high as 10^6^-fold and depends on the synergistic production of tumor necrosis factors (13, 16). In this assay, 4 h after the i.v. administration of any superantigen, sublethal amounts of LPS from Salmonella enterica serovar Typhimurium are given i.v. Animals are then monitored for up to 48 h for lethality. The relationship of pyrogenic toxin superantigen pretreatment with subsequent LPS challenge for a 50% lethal dose (LD_50_) endpoint is log-log such that for every log increase in pyrogenic toxin superantigen administered, there is a log decrease in the concentration of LPS required for 50% lethality (13). Pyrogenic toxin superantigens must be administered prior to LPS administration (13). The LD_50_ of LPS alone intravenously in rabbits is approximately 500 μg/kg (13). The LD_50_ of superantigens alone intravenously in rabbits is >5.0 mg/kg. However, for example, the preadministration of 1.0 μg/kg of TSST-1 to rabbits requires 0.05 μg/kg of LPS to cause the deaths of 50% of animals. Because death as an endpoint is no longer acceptable, the Schlievert laboratory now uses two other criteria in this assay, which predict uniform lethality: (i) when rabbits cannot sit erect and, at the same time, (ii) when they do not exhibit normal escape behavior from humans. It is important to emphasize that both pyrogenicity and the enhancement of susceptibility to LPS shock are measurable in the same rabbits, usually three per group. The final pyrogenic toxin superantigen assay used in the Schlievert laboratory is splenocyte proliferation *in vitro* where maximum splenocyte and, therefore, T cell proliferation occurs after a 4-day incubation with pyrogenic toxin superantigen concentrations of 10^−8^ to 10 μg/2 × 10^5^ splenocytes. Proliferation is measured by the incorporation of [^3^H]thymidine into DNA (17). For TSST-1, SEB, and SEC, statistically significant splenocyte proliferation occurs at ≥10^−5^ μg/2 × 10^5^ splenocytes.

Previously, we and others have studied the vaccination of rabbits and humans against TSST-1 by the use of vaccine toxoids (18–20). TSST-1 mutants that lack the ability to cause fever and enhance LPS shock and that fail to stimulate T cell proliferation can also be used to vaccinate rabbits against infective endocarditis and consequent sepsis as well as pneumonia (21, 22). This depends on making mutants of protein amino acids that conserve the overall three-dimensional structure while mutating immune cell contact residues. Similar types of studies have also allowed us to prepare toxoid vaccines against two major streptococcal pyrogenic exotoxin (SPE) superantigens (23, 24), including SPE A, which shows 50% sequence similarity to SEs B and C (23).

The goal of this study was to prepare suitable toxoid vaccines against SEB and SEC, which, in addition to TSST-1, are the major causes of staphylococcal TSS. These studies were performed in part prior to SEs being referred to as select agents and when my laboratory was a Centers for Disease Control and Prevention (CDC)-approved laboratory for select-agent studies at the University of Minnesota. Plasmids encoding either wild-type SEB or SEC have been considered select agents of bioterrorism since 2001. Subsequently, the CDC approved the use of plasmids with *seb* and *sec* mutations (Q210A and N23A) without these toxoids being considered select agents. Until their approval by the CDC for standard biosafety level 2 (BSL2) use, mutant clones were stored without use in a safe by the Schlievert laboratory, as required by the Department of Environmental Health and Safety.

This study shows that SEB and SEC mutants (N23A, Q210A, and N23A/Q210A) lacked biological toxicity in the three assays used in the laboratory to assess toxicity. These mutants protect animals in multiple models of toxicity, and they can be combined to make additional toxoids. Because of multiple amino acid mutations, the resultant toxoids are unlikely to revert to having toxicity.

## RESULTS

### Select-agent and animal use requirements.

All experiments were performed according to CDC requirements for the use of select agents of bioterrorism for native SEB and SEC proteins and their genes on plasmids. Wild-type SEB and SEC were maintained at concentrations of <5 mg, as originally required by the CDC, per total combined amounts of SEs stored in a safe accessible in the laboratory by only P. M. Schlievert. Later, the CDC raised the SE select-agent exemption level to 100 mg total. The *seb* gene cloned into plasmid pMIN164 with expression in Staphylococcus aureus RN4220 was used prior to the plasmid being referred to as a select agent to generate SEB mutant Q210A by the site-specific mutagenesis of wild-type *seb*. Similarly, SEC N23A was prepared prior to the plasmid containing wild-type *sec* being referred to as a select agent. Just prior to the Schlievert laboratory stopping select-agent laboratory designation, all wild-type *seb* and *sec* plasmid clones were destroyed by autoclaving. The plasmids expressing SEB Q210A and SEC N23A were then stored at −80°C as lyophilized stocks until the CDC allowed their use as nonselect agents. Once these plasmids were deregulated, additional mutations were made to generate SEB N23A, SEB N23A/Q210A, SEC Q210A, and SEC N23A/Q210A.

All animal experiments were performed with Institutional Animal Care and Use Committee (IACUC) approval from the University of Minnesota and the University of Iowa, as applicable. Dutch Belted rabbits, both male (60%) and female (40%) and weighing 1 to 2 kg, were used for these studies. More males than female rabbits were used because of the retention of breeding stocks by the provider. Rabbits were purchased from Bakkom Rabbitry, Red Wing, MN.

### Superantigenicity of SEB and SEC mutants compared to wild-type proteins.

**FIG 1:**
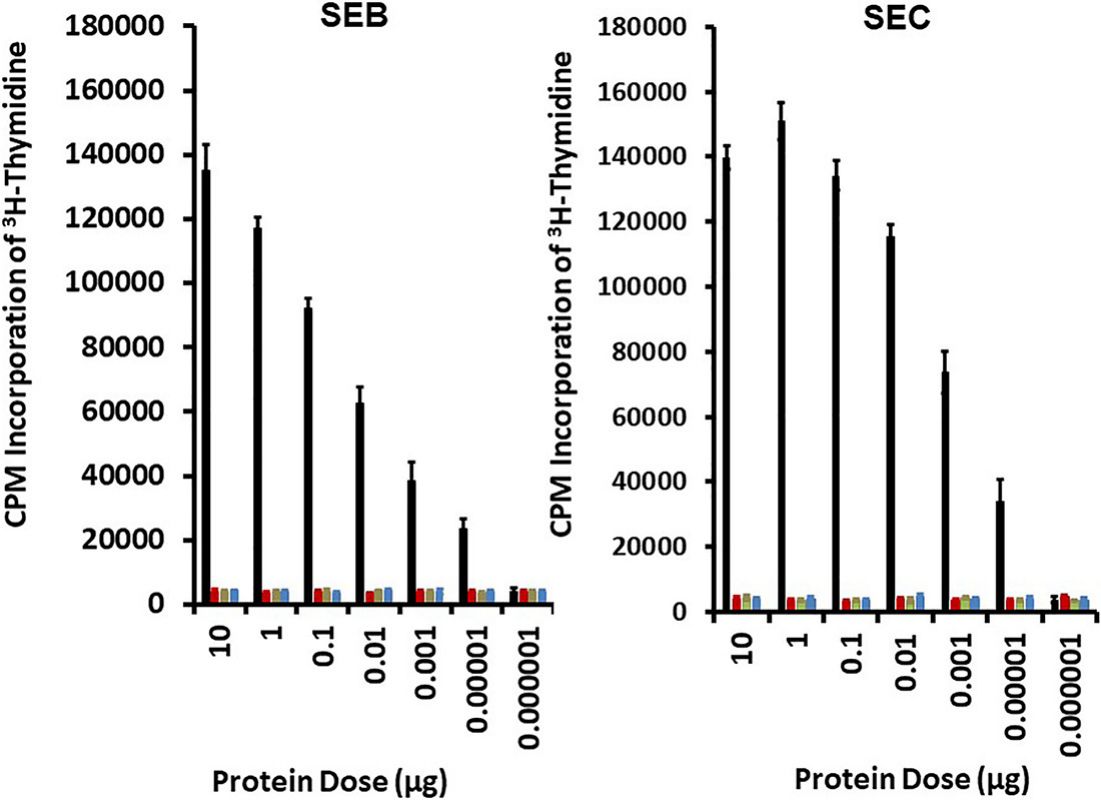
Superantigenicity as measured by the proliferation of rabbit splenocytes of wild-type SEs (black bars) and N23A (red bars), Q210A (gray bars), and N23A/Q210A (blue bars) mutants. Proliferation was measured by the incorporation of [^3^H]thymidine into DNA in counts per minute (CPM) after 4 days of incubation with SEB, SEC, or mutants of SEB or SEC. Thymidine (1 μCi) was added 24 h before DNA was harvested. Assays were performed in quadruplicate 96-well culture plates with 2 × 10^5^ splenocytes/well in 200 μL complete RPMI 1640 medium. Wild-type and mutant SE concentrations per well ranged from 10^−6^ to 10 μg/well. Four control wells contained only splenocytes in 200 μL complete RPMI 1640 medium. Values reported are means ± standard deviations. *P* values for significant differences in means between wild-type SEs and the same doses of the mutants were <0.001 for all doses from 10 μg/well to 0.00001 μg/well. The *P* value was not significant for wild-type versus mutant SEs, at 0.000001 μg/well.

At all doses of SEB N23A, SEB Q210A, SEB N23A/Q210A, SEC N23A, SEC Q210A, and SEC N23A/Q210 tested, the proteins lacked statistically significant superantigenicity as measured by the proliferation of rabbit splenocytes (see Fig. 1A for SEB and Fig. 1B for SEC). In contrast, wild-type SEB and SEC caused significant superantigenicity at all doses from 10 μg/2 × 10^5^ splenocytes to 10^−5^ μg/2 × 10^5^ splenocytes. The data showed that all six mutant SEs lost at least 6 logs of superantigenicity, one of the most sensitive assays for toxicity. Also, importantly, both double mutants lacked superantigenicity, and if there was happenstance reversion to wild-type SE in one amino acid position, the proteins would still lack superantigenicity.

### SEB and SEC mutant enhancement of endotoxin (LPS) shock.

The most sensitive *in vivo* measure of the toxicity of SEB and SEC is the ability of the wild-type SEs to enhance LPS shock. This enhancement can be as high as 10^6^-fold (13). When 1,000 μg/kg of either wild-type SEB or SEC was administered to rabbits i.v., there was no lethality observed in the rabbits over a 48-h time period (Table 1). The LD_50_ of LPS alone varied from 500 μg/kg to 550 μg/kg in rabbits. However, when 1,000 μg/kg of either SEB or SEC was administered i.v. to rabbits, the LD_50_ of LPS was approximately 0.0008 μg/kg. The LD_50_ of LPS following the administration of any of the three mutants of SEB or SEC (1,000 μg/kg) was >100 μg/kg (the highest LPS dose tested), indicating that the six SE mutants individually lacked the ability to enhance endotoxin shock. They were thus >10^5^ inactivated in the enhancement phenomenon.

**TABLE 1:** Enhancement of LPS-induced shock by 1,000 μg/kg of wild-type SEB and SEC or 1,000 μg/kg of mutants^*a*^

Treatment	LD_50_ of LPS (μg/kg)
Wild-type SEB	0.0008
SEB N23A	>100
SEB Q210A	>100
SEB N23A/Q210A	>100
Wild-type SEC	0.0008
SEC N23A	>100
SEC Q210A	>100
SEC N23A/Q210A	>100

a Note that there was no measurable LD_50_ of intravenous wild-type SEB or SEC alone by this method. The LD_50_ of intravenous Salmonella enterica serovar Typhimurium LPS alone varied between 500 μg/kg and 550 μg/kg.

### SEB and SEC mutant pyrogenicity.

Prior to being referred to as superantigens, the large family of SEs, TSST-1, and SPEs was referred to as pyrogenic toxins (15). This was based on their ability to cause high fevers in rabbits and diseases characterized by high fevers in humans (4, 25, 26). The wild-type and mutant SEB and SEC proteins were tested for pyrogenicity in the standard assay after the i.v. injection of 100 μg/kg, 10 μg/kg, and 1 μg/kg to three rabbits per dose. All superantigens cause fevers that peak 4 h after injection. The minimum pyrogenic dose after 4 h (MPD-4) was determined as the dose required to cause an average fever response in three rabbits at the 4-h time point of 0.5°C.

**FIG 2:**
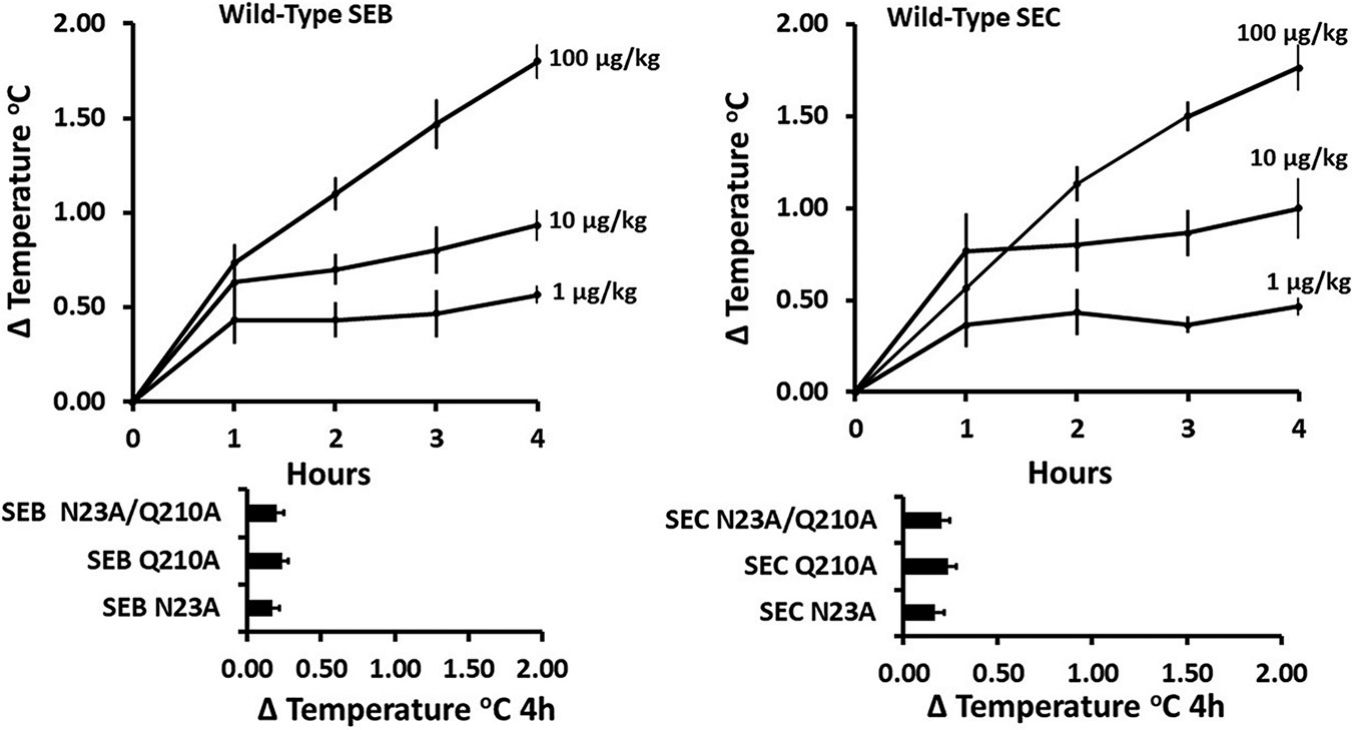
Pyrogenicity of wild-type SEs B and C over a 4-h time period and 4-h peak pyrogenicity of six mutants of SEB and SEC (N23A, Q210A, and N23A/Q210A). Values reported are mean changes in body temperatures of 3 rabbits/group ± standard deviations. Doses of wild-type SEs were 100 μg/kg, 10 μg/kg, and 1 μg/kg. Data for mutant SEs are shown only for the 100-μg/kg dose.

Pyrogenicity curves are shown in Fig. 2. Wild-type SEB and SEC were pyrogenic in rabbits, with MPD-4/kg values of approximately 1 μg/kg for both SEB and SEC. In contrast, none of the six mutant proteins (SEB N23A, SEB Q210A, SEB N23A/Q210A, SEC N23A, SEC Q210A, and SEC N23A/Q210A) were pyrogenic at the highest dose tested (100 μg/kg). Thus, the mutant proteins were >100-fold inactivated for the ability to cause fever.

### Vaccination of rabbits against wild-type SEB and SEC and SEB- or SEC-producing S. aureus.

First, five rabbits per group were vaccinated three times with each mutant of SEB and SEC. After resting for 1 week, a small sample of blood was drawn from the left marginal ear vein of each animal for the assessment of the antibody response. Next, 1 week later (2 weeks after the last booster vaccination) or after 3.5 months, the rabbits were challenged in the endotoxin enhancement model i.v. with wild-type SEB or SEC (10 μg/kg), followed by LPS (10 μg/kg) at 4 h; this combination of wild-type SE and LPS would typically reduce the LD_50_ of LPS to 0.05 μg/kg (10,000-times reduction in the LPS LD_50_). Five rabbits per group remained unvaccinated.

**FIG 3:**
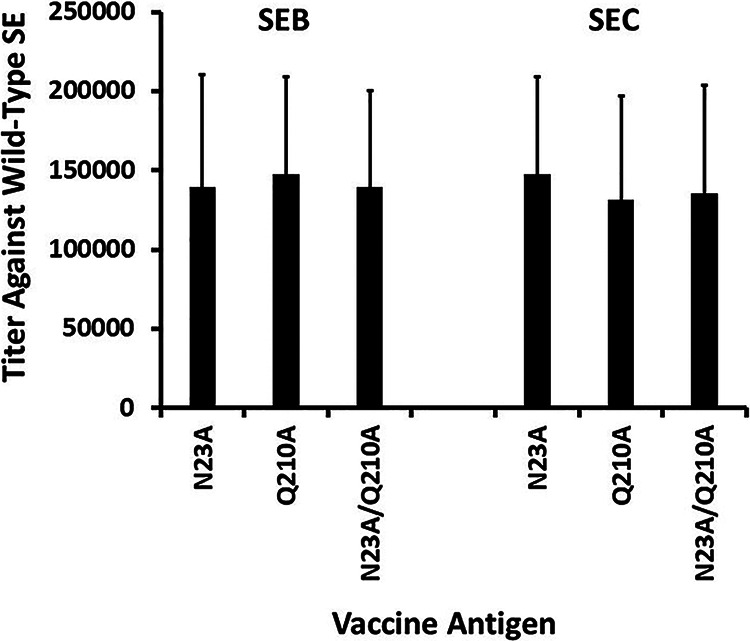
ELISA antibody titers of 10 rabbits/group vaccinated against the indicated antigen tested against homologous wild-type SEB or SEC. Bars indicate standard deviations of titers. Titers were the reciprocal of the dilution of the last well showing a positive reaction. The range in titers of vaccinated animals was 40,960 to 245,760. Rabbits were evaluated for antibody titers 1 week after the third vaccination.

The titers of antibodies to the homologous SE after vaccination, as assessed by the use of an enzyme-linked immunosorbent assay (ELISA) and plates coated with wild-type SE, are shown in Fig. 3. The titers in vaccinated animals, regardless of the vaccine antigen, exceeded 10^5^. In contrast, the preimmune titers were <10, with 10 being the lowest titer assayed; lower dilutions of sera were not evaluated because of interference due to nonspecific reactions.

For the rabbits vaccinated with the mutant SEs, none showed fevers due to wild-type SE challenge over a 4-h period, and none succumbed to lethal challenge with the homologous wild-type SE followed by LPS, whether 2 weeks after the last vaccination or 3.5 months after the last vaccination (Table 2). In contrast, all nonvaccinated rabbits showed fever responses due to challenge with wild-type SE, and all succumbed to enhanced susceptibility to LPS shock. For each vaccine antigen, the *P* value for the mean difference between lethality in vaccinated and nonvaccinated animals was 0.008 by Fisher’s exact test. Data from animals vaccinated against the double mutants were combined with data from the single mutant-vaccinated animals since the double mutant groups were also immune to the respective single mutants. The *P* values by Fisher’s exact tests were then 0.0003. These data suggested that the vaccine antigens were highly effective in preventing the toxicity of homologous wild-type SEs B and C.

**TABLE 2:** Vaccine protection of rabbits from challenge with homologous wild-type SEB or SEC

Vaccine antigen	Time tested	Mean fever response 4 h after i.v. injection (°C) ± SD	No. of dead rabbits/5 total due to LPS enhancement
None^*a*^	2 wks	1.1 ± 0.05	5
None^*a*^	3.5 mo	1.2 ± 0.0	5
SEB N23A^*a*^	2 wks	0.3 ± 0.05	0
SEB N23A^*a*^	3.5 mo	0.3 ± 0.05	0
SEB Q210A^*a*^	2 wks	0.4 ± 0.05	0
SEB Q210A^*a*^	3.5 mo	0.3 ± 0.05	0
SEB N23A/Q210A^*a*^	2 wks	0.2 ± 0.05	0
SEB N23A/Q210A^*a*^	3.5 mo	0.3 ± 0.05	0
None^*b*^	2 wks	1.2 ± 0.1	5
None^*b*^	3.5 mo	1.3 ± 0.1	5
SEC N23A^*b*^	2 wks	0.3 ± 0.05	0
SEC N23A^*b*^	3.5 mo	0.2 ± 0.05	0
SEC Q210A^*b*^	2 wks	0.3 ± 0.05	0
SEC Q210A^*b*^	3.5 mo	0.3 ± 0.05	0
SEC N23A/Q210A^*b*^	2 wks	0.4 ± 0.05	0
SEC N23A/Q210A^*b*^	6.5 mo	0.2 ± 0.05	0

a Challenged with wild-type SEB plus LPS after vaccination.

b Challenged with wild-type SEC plus LPS after vaccination.

**FIG 4:**
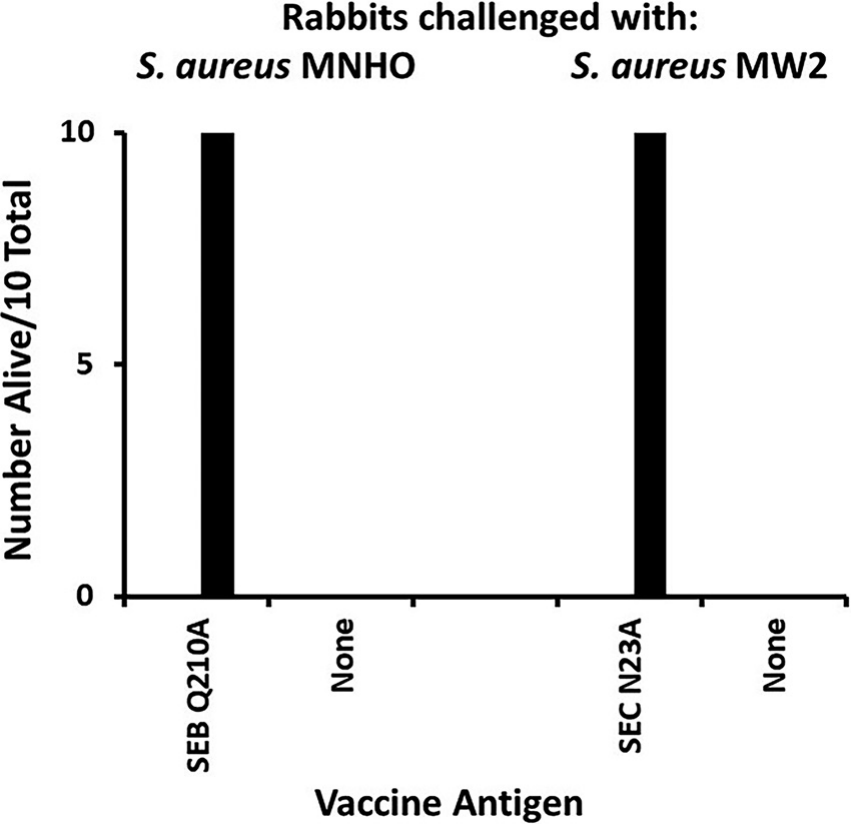
Protection of rabbits (number alive/10 total) from S. aureus strains MNHO (produces SEB) and MW2 (produces SEC) by vaccination with either SEB Q210A or SEC N23A or in nonvaccinated animals (None). The *P* value by Fisher’s exact test for immunized versus nonimmunized animals was 0.00001.

Groups of rabbits (5/group performed twice, for a total of 10/group) were also immunized with either SEB Q210A or SEC N23A, or remained nonvaccinated, and then challenged with native methicillin-resistant S. aureus strain MNHO (SEB) or MW2 (SEC) in a rabbit pneumonia model (Fig. 4). All rabbits were challenged intratracheally with approximately 3.0 × 10^9^ CFU of S. aureus suspended in 0.4-mL volumes. The animals were monitored for 7 days for lethality. All vaccinated animals survived, whether vaccinated against SEB Q210A or SEC N23A. These animals showed low-grade fevers (0.5°C to 1.0°C) for 2 to 3 days, but examination of their lungs on day 7 showed healthy or healing lungs. In contrast, nonvaccinated rabbits showed high fevers (>2.0°C), and all succumbed in <4 days. Their lungs showed extensive hemorrhagic pneumonia. The *P* value for the death of vaccinated versus nonvaccinated rabbits for each challenge organism was 0.00001 by Fisher’s exact test, indicating that these two vaccines were highly effective in preventing lethality.

## DISCUSSION

There are three major causes of staphylococcal TSS (1–3). TSST-1 is the cause of 100% of menstrual TSS and 50% of nonmenstrual TSS cases (3). SEB and SEC cause nearly 50% of nonmenstrual TSS cases (1, 2). All three of these pyrogenic toxin superantigens are likely important in many other S. aureus infections. For example, TSST-1 is highly associated with eczema herpeticum (27) and the blistering disease bullous pemphigoid (28). SEs B and C are highly associated with necrotizing pneumonia associated with USA400 clonal group strains (29), including MNHO (SEB) and MW2 (SEC) as I studied here. The latter two pyrogenic toxin superantigens are also causes of staphylococcal food poisoning (30).

Vaccine toxoids of TSST-1 have been prepared, involving alterations of surface amino acid residues that interact with MHC II and/or Vβ2-TCR (18, 19, 31). It is my experience in collaboration with other researchers in the evaluation of large numbers of mutants of TSST-1, SEB, and SEC that alteration of surface amino acids does not change the overall domain three-dimensional structure of these pyrogenic toxin superantigens (10, 11, 19, 23, 32–45). Thus, the resultant mutants could be considered toxoids, which may induce immunity that protects against wild-type toxins.

A phase I clinical trial has been performed, which documented the safety of and human antibody responses to a toxicity-inactivated TSST-1 mutant (31). These data are significant since it is known that as many as 20% of humans over the age of 12 years are unable to develop protective antibodies against wild-type TSST-1 (46–48). The fact that all persons responded with antibody production in the trial suggests that persons who do not develop antibodies to wild-type TSST-1 respond too aggressively to the toxin-induced superantigen response, preventing antibody production. I have previously suggested this lack of an antibody response in 20% of humans results from the overproduction of interferon gamma, which then suppresses antibody production (49).

There are multiple studies that have identified amino acid residues on SEB and SEC that are required for superantigenicity (11, 35, 37, 50, 51). These previous studies provide the basis for studies in the current research. Prior to SEB, SEC, and the plasmids encoding them being categorized as select agents, the Schlievert laboratory prepared mutant proteins through the site-specific mutagenesis of the plasmid-contained genes *seb* and *sec*. These mutants were then shelved until the CDC deregulated their use because of the demonstrated lack of toxicity. However, even though the data were shared with the CDC, these vaccine studies have not previously been published. Thus, the current research is now presented.

It would have been interesting to study mutants of SEB and SEC that have partial activities. Such mutants were isolated and shown to at least have intermediate superantigenicity prior to their becoming regulated as select agents (11). No further work has been done in my laboratory with these mutant proteins. However, we have shown that at least 4 of the pyrogenic toxin superantigen activities may be separable. For example, SEB and SEC are emetic in monkeys, whereas TSST-1 is not emetic (30). This difference appears to be due in part to differences in mucosal barrier penetration by these toxins. Additionally, recent evidence indicated that pyrogenic toxin superantigens interact with the immune costimulatory molecule on epithelial cells and keratinocytes to elicit surface inflammation (7, 8). Finally, a study using monoclonal antibodies to TSST-1 suggested that even the abilities to cause fever and enhance endotoxin shock appear separable (52).

Prior to being named superantigens, this large family was referred to as pyrogenic toxins (15). This was based on the impressive ability of the proteins to cause fever (12). Indeed, the SPEs produced by Streptococcus pyogenes, which are the causes of severe scarlet fever, are often considered the most potent pyrogens known (1, 2). As shown in this study, SEB and SEC, which show 50% sequence similarity to SPE A (53, 54), are potent pyrogens. In contrast, the mutants prepared in this study lacked the ability to cause fevers in our standard model of rabbit pyrogenicity. With the doses used, the current studies indicate that all six mutants, three for SEB and three for SEC, were at least 100-fold inactivated for their ability to induce fevers.

Another major shared biological activity that helped define pyrogenic toxins is their ability to enhance lethal endotoxin shock by up to 10^6^-fold (13, 55). It has been proposed that this activity explains the lethality of these proteins, as evidenced by toxicity to rabbits and humans but much less so to mice, which are highly resistant to LPS lethal activity (56). In the current studies, it was shown that all six mutants lacked a demonstrable enhancement of LPS shock. The data showed that all of the mutants were at least 10^5^-fold inactivated, essentially the lower limit of the assays.

The abilities of pyrogenic toxin superantigens to cause fever and enhance LPS shock depend on cytokine production by proliferating and/or activated immune cells, notably interleukin-1β from macrophages and tumor necrosis factors alpha and beta from macrophages and T lymphocytes (16, 57). The current study showed that all six SE mutants lacked superantigenicity as measured by rabbit splenocyte proliferation. The degree of inactivation was >10^6^-fold, essentially the limit of the assay.

Collectively, the three bioassays described above that were used to evaluate toxicity indicate that the mutants of SEB and SEC are nontoxic and have the potential for use as toxoid vaccines.

The final property that studies in this research investigated was the ability of the mutant, nontoxic SEs to immunize rabbits against lethal challenge by homologous wild-type pyrogenic toxin superantigens and SEB- or SEC-producing methicillin-resistant S. aureus. After three vaccinations (one primary and two boosters), the rabbits showed average antibody titers against homologous SEs of >100,000. Additionally, the rabbits were protected from uniform lethal challenge in the LPS enhancement model. Although already highly significantly protected compared to unvaccinated animals, the data become even more significant when protection data are combined for the mutant proteins as follows: (i) N23A plus N23A/Q210A and (ii) Q210A plus N23A/Q210A. The *P* values by Fisher’s exact test become <0.0003 instead of <0.008. If the data for rabbits tested 2 weeks after vaccination are combined with the data from rabbits challenged 3.5 months after vaccination, the *P* value becomes <0.0001. In one final set of studies, rabbits were vaccinated against two of the single-site mutants (SEB Q210A or SEC N23A). After vaccination, all of the rabbits were protected from lethal pneumonia (nonmenstrual TSS) due to two different USA400 (clonal complex 1 [CC1]) methicillin-resistant S. aureus strains, one producing SEB and one producing SEC. These data collectively suggest that the SEB and SEC mutants should be considered important vaccine toxoids that could prevent cases of nonmenstrual staphylococcal TSS.

Where would the TSST-1, SEB, and SEC toxoids be used for vaccination? Increasingly, staphylococcal infections are being recognized for lethality due to wild-type TSST-1, SEB, and SEC. For example, highly lethal postinfluenza TSS due to TSST-1 may have accounted for as many as 50,000 deaths in children in the United States since the description of the infection (4). These data are obtained by calculating that approximately 10 to 15 children succumbed to TSST-1-induced postinfluenza TSS in the Minneapolis-St. Paul area in 1987 (4). If these numbers are extended to the entire United States from 1987 to the present, the number 50,000 is obtained. It is also clear that SEB and SEC cause nonmenstrual TSS (4, 5). For example, it is known that all USA400 (CC1) strains produce either SEB or SEC (29, 58). These strains continue to cause fatal TSS in the form of necrotizing pneumonia, particularly in the Midwestern United States (59–61). Finally, SEB and SEC are select agents of bioterrorism. This is in part based on the observation that the United States has long recognized the toxicity of these proteins. Indeed, SEB was included as the number 1 bioweapon in the United States, where the country stockpiled as much as 5 to 6 tons per year in the 1950s and 1960s (62). SEs have the potential to be fatal at concentrations of as low as 0.1 to 1.0 μg/human (63). Additionally, concentrations of as low as 1 ng may be able to cause 1 to 2 days of vomiting and diarrhea by the oral route (30, 64). Finally, even doses below the nanogram range may induce red eyes in exposed persons (https://www1.nyc.gov/site/doh/health/health-topics/staphylococcal-enterotoxin-b.page). Evaluation of protection against the latter activities was beyond the scope of the current study, but the activities and possible protection should be evaluated in the future.

## MATERIALS AND METHODS

### Bacteria and clones.

Staphylococcal strain MNHO was the source of SEB (65). MNDon was the source of SEC (66). These were clinical isolates from patients who met the criteria for nonmenstrual TSS. The strains of low passage number are stored at −80°C. Both MNHO and MW2 are methicillin-resistant USA400 (CC1) S. aureus strains. Both strains came from patients who met the criteria for nonmenstrual TSS and/or hemorrhagic pneumonia.

Clones expressing SEB and SEC mutants were prepared prior to the plasmids being considered select agents of bioterrorism, first prepared in Escherichia coli and then transferred to S. aureus RN4220, as my laboratory has done for other pyrogenic toxin superantigen genes (18). The methods for preparing the mutants have been described previously (11). Briefly, the gene for SEB was generously provided by C. Jones and S. Khan (University of Pittsburgh School of Medicine, Pittsburgh, PA). Cloning and sequencing of the *sec* gene were reported previously by Hovde et al. (67). SEB and SEC mutants were produced by site-directed mutagenesis (18). Mutagenic oligonucleotides were designed to replace codons for asparagine and glutamine with codons for alanines in the SEB and SEC genes. The mutations were confirmed by DNA sequencing. Site-specific mutagenesis was performed prior to the plasmids being considered select agents to prepare single and double mutations in both the SEB and SEC genes.

### Production of SEs B and C and mutants.

Wild-type SEB was produced from MNHO, and wild-type SEC was prepared from MNDon. Cultures of each organism were grown in dialyzed Todd-Hewitt broth medium (1.0 L per organism) at 37°C, with high aeration (shaking at 200 rpm), until stationary phase was achieved (18 to 24 h) (68). At this time, the cultures were treated with absolute ethanol to achieve 80% (vol/vol) to precipitate enterotoxins. After sitting for 48 h at 4°C, the precipitates were collected by centrifugation (4,000 × *g* for 15 min), the enterotoxins were solubilized by the addition of 100 mL sterile pyrogen-free distilled water, and enterotoxins were purified by preparative thin-layer isoelectric focusing. Both SEB and SEC migrated with a major protein band at pH 8.0 (68).

Mutant proteins were prepared from S. aureus RN4220 clones after growth and purification as described above for wild-type enterotoxins, except that S. aureus with mutant genes was cultured in the presence of 5 μg/mL erythromycin.

### Bioassays.

Three assays were performed to assess the biological toxicity of mutant SEs B and C compared with wild-type enterotoxins: (i) *in vitro* superantigenicity with rabbit splenocytes (17), (ii) pyrogenicity in rabbits (12), and (iii) the ability to enhance the susceptibility of rabbits to lethal endotoxin shock (13). Briefly, for superantigenicity (17), splenocytes were extracted from rabbit spleens by forcing liquid from syringes with 20-gauge needles into the spleens. The splenocytes were counted and placed into 96-well tissue culture wells at 2 × 10^5^ splenocytes/200 μL of complete RPMI 1640 medium. Next, 20 μL of serial 10-fold dilutions of wild-type and mutant enterotoxins was added in quadruplicate. The plates were then incubated for 3 days in a 5% CO_2_ incubator, after which time 1 μCi [^3^H]thymidine was added to each well. The plates were incubated for an additional 24 h, and DNA from proliferating splenocytes (presumably T lymphocytes) was harvested onto glass fiber filters. [^3^H]thymidine collected onto glass fiber filters was assessed by scintillation counting, recording the counts per minute. It is important to emphasize that pyrogenic toxin superantigens, such as SEB and SEC, cause splenocyte proliferation that peaks 4 days after the addition of SEs. Thus, this assay was assessing maximum [^3^H]thymidine incorporation.

The fever responses of rabbits to pyrogenic toxin superantigens, including SEB and SEC, rise relatively linearly, peaking 4 h after i.v. administration in sterile, pyrogen-free saline (12). Three doses (100 μg/kg, 10 μg/kg, and 1 μg/kg) of each protein were administered i.v. to rabbits, and changes in body temperature were determined by the use of rectal thermometers. The minimum pyrogenic dose per kilogram (MPD-4/kg) was determined as the concentration of SEB, SEC, or mutant proteins needed to cause an average (3 rabbits/dose) rise in body temperature of 0.5°C at the 4-h time point.

The ability of SEB and SEC to enhance rabbit susceptibility to LPS-induced shock was measured 4 h after the i.v. administration of wild-type and mutant enterotoxins (13). For this assay, 3 rabbits/group were administered 1,000 μg/kg of wild-type or mutant SEBs and SECs at time zero by i.v. injection. These are not lethal doses alone of SEB or SEC. Subsequently, at 4 h, Salmonella Typhimurium LPS was injected i.v., and the LD_50_ was determined by recording deaths over a 48-h time period. Since death as an endpoint is not allowed, in agreement with university IACUC protocols, death was recorded as the simultaneous failure of rabbits to be able to remain upright and to exhibit typical escape behavior from humans. This point has been shown to be 100% predictive of lethality in this assay.

For intrapulmonary administration, organisms (MNHO and MW2) were grown overnight in 25 mL of Todd-Hewitt broth (Difco Laboratories, Detroit, MI) at 37°C with shaking at 200 rpm. The organisms were washed once with phosphate-buffered saline (PBS) (0.005 M NaPO_4_ [pH 7.2], 0.15 M NaCl), followed by centrifugation at 20,800 × *g* for 5 min, and then resuspended in Todd-Hewitt broth at 3.0 × 10^9^ CFU/0.4 mL for high-dose injection.

Rabbits were administered bacteria via intratracheal inoculation (21). Rabbits were first anesthetized with ketamine (25 mg/kg) and xylazine (25 mg/kg) (Phoenix Pharmaceuticals, Burlingame, CA). Their necks were shaved, and small incisions were made to expose the tracheas. Incisions were made into the tracheas, followed by insertions of 1-mm-diameter polyethylene catheters (Becton, Dickinson and Co.), threading them into the left bronchi. Microbes were administered through the catheters, the catheters were removed, and the incision sites were closed. Rabbits were monitored for 7 days for the development of pneumonia, TSS symptoms (fever, difficulty breathing, diarrhea, reddening of conjunctivae, and evidence of hypotension), and lethality. Rabbits were prematurely euthanized when they simultaneously failed to remain upright and could not exhibit flight responses expected of wild rabbits when approached by humans. Animals were euthanized with 1 mL/kg of pentobarbital sodium and phenytoin sodium (Euthasol; Virbac, Westlake, TX). The surviving rabbits were euthanized at the end of 7 days, and their lungs were examined for gross damage.

### Vaccinations.

Mutant SEs B and C were used to vaccinate Dutch Belted rabbits (1 to 2 kg). The proteins (50 μg/injection) were emulsified in Freund’s incomplete adjuvant and administered to rabbits subcutaneously in the napes of the necks every other week for three injections (21, 22). Prior to the initiation of the vaccination schedule and 1 week after the last injection, a small amount (0.5 mL) of blood was removed through the marginal ear veins to determine the ELISA titers of antibodies to the respective proteins. The titer was defined as the reciprocal of the last positive reaction in 96-well microtiter plates.

An ELISA was performed as described previously (69), Briefly, 1.0 μg/well of either wild-type SEB or SEC was used to coat the wells of 96-well Immulon ELISA plates (Thermo Fisher). Since the only reactions of interest were reactions of antibodies to wild-type SEs B and C, only wild-type SEB or SEC was used to coat the wells. After the addition of a well-site-blocking agent, serial 2-fold dilutions (beginning with a dilution of 1:10) of serum from preimmune or postimmunized rabbits were made across the wells. The plates were incubated for 2 h at room temperature. Next, after the washing steps, peroxidase-conjugated anti-rabbit IgG (1/1,000 dilution) was added to each well. After an additional incubation for 2 h at room temperature, the wells were again washed, and the substrate was then added. After color development, sulfuric acid was used to stop the reactions. Each plate was then read on an ELISA plate reader.

### Statistics.

Means ± standard deviations (SDs) were determined. Differences in means for superantigenicity and pyrogenicity were determined by Student’s *t* test of normally distributed, unpaired data. Differences in lethality were determined by Fisher’s exact test.
